# Yemen: Fighting Neglected Tropical Diseases against All Odds

**DOI:** 10.1371/journal.pntd.0003292

**Published:** 2014-12-18

**Authors:** Elisa Baring, Peter J. Hotez

**Affiliations:** 1 The END Fund, New York, New York, United States of America; 2 Department of Pediatrics and Molecular Virology and Microbiology, National School of Tropical Medicine, Baylor College of Medicine, Houston, Texas, United States of America; 3 Sabin Vaccine Institute and Texas Children's Hospital Center for Vaccine Development, Houston, Texas, United States of America; 4 James A Baker III Institute for Public Policy, Rice University, Houston, Texas, United States of America; 5 Department of Biology, Baylor University, Waco, Texas, United States of America

**Figure 1:**
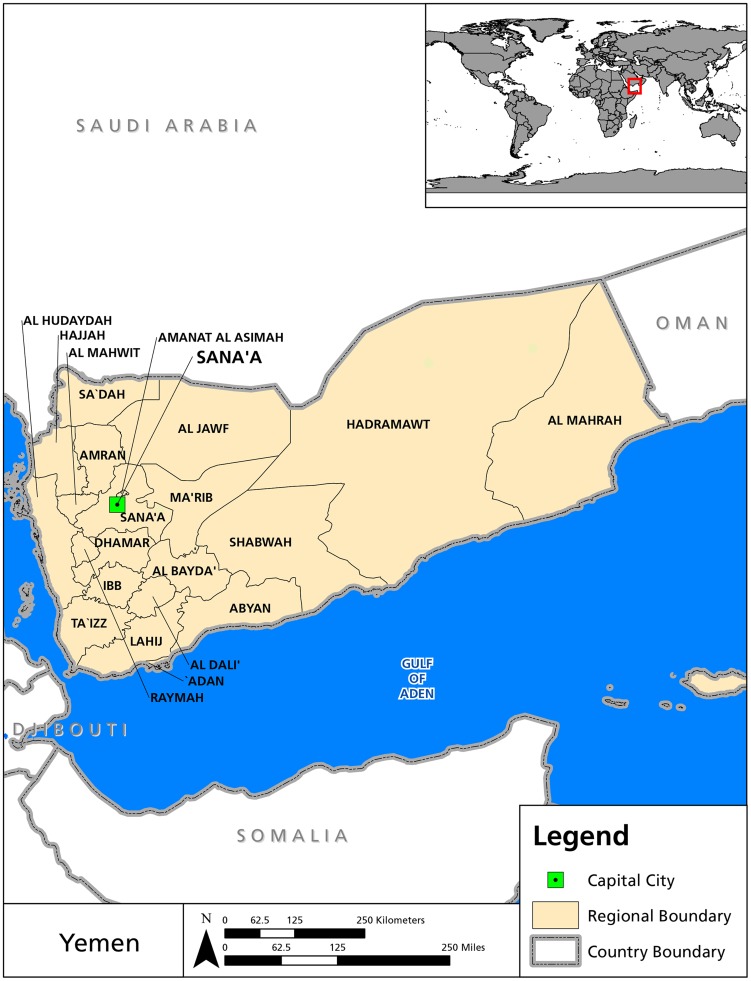
Map of Yemen. (Original figure.)

Yemen is a low-income country on the Arabian Peninsula (Fig. 1) with a human development index equivalent to that of Nigeria or Madagascar [1]. It is also a nation beset by violence because of a southern secessionist movement and, most recently, an escalating level of civil unrest in the capital of Sana′a [2]. In addition to rising levels of violence fueled by ongoing tribal conflicts and the presence of an Al-Qaeda insurgency, the country is faced with rising levels of unemployment (estimated overall unemployment is 17.34% [3] and youth unemployment is 34.8% [4]) and poverty (52.5% of the population live in multidimensional poverty [5]), and a growing youth population (in 2012 the fertility rate was 4.2 and the annual population growth rate was 3.2 [6]).

As we have seen previously in some sub-Saharan African countries, such as South Sudan and Central African Republic, neglected tropical diseases (NTDs) are known to flourish in this setting of poverty and instability. Today, Yemen has some of the highest concentrations of NTDs in the Middle East [7]. According to the World Health Organization (WHO), more than one-third of the people of Yemen require regular treatment for schistosomiasis [8], an important cause of urogenital disease, especially among girls and women (who develop female genital schistosomiasis), as well as bladder cancer (Table 1). More than 10 million Yemeni children require treatment for intestinal worms, which cause malnutrition and cognitive delays [9]. River blindness and trachoma, each requiring preventive chemotherapy, are endemic [7],[10], as are cutaneous leishmaniasis and dengue fever [11],[12].

**Table 1:** The major neglected tropical diseases of Yemen.

Disease	Estimated number of people requiring treatment or number of cases	Reference
Schistosomiasis	9.1 million people, including 2.9 million school-aged children	[8]
Intestinal helminth infections	3.4 million preschool-aged children	[9]
	6.9 million school-aged children	
Onchocerciasis	Not determined	[2]
Trachoma	>200,000 cases	[7]
Cutaneous Leishmaniasis	3,000 to 6,000 cases	[11]
Dengue	222,930 apparent cases	[12]
	689,860 inapparent cases	

An important reason why we need to care about NTDs is that these conditions are often chronic and debilitating and actually cause or reinforce poverty through their long-term effects on children and heads of households. They disproportionately strike girls and women. As a result, NTDs can also destabilize communities and possibly even further help to promote conflict [13]. Thus, NTD control and elimination represent important critical steps to help stabilize the nation of Yemen and to promote its economic development.

**Figure 2:**
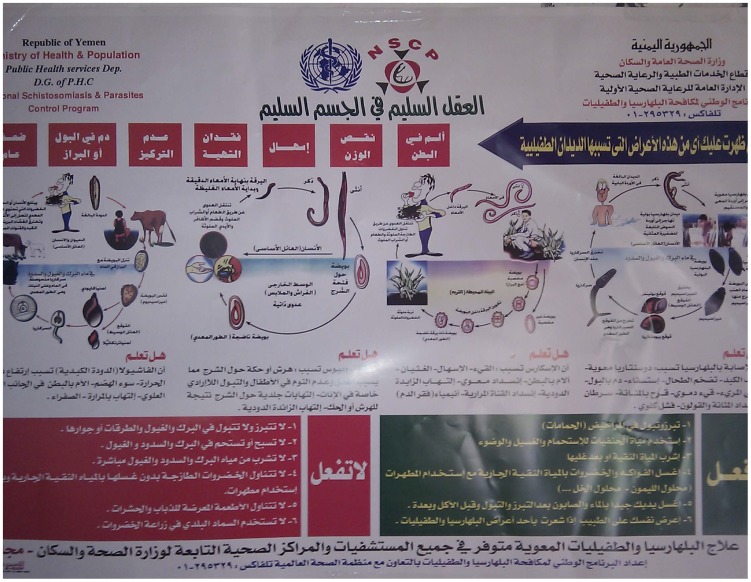
Schistosomiasis treatment campaign. 2010 informational poster from Yemen's National Schistosomiasis Control Program depicting the disease transmission cycle for schistosomiasis and providing information about both prevention and treatment.

In late 2009, the World Bank approved an important project (the Yemen Schistosomiasis Project [YSP]) to eliminate schistosomiasis-related morbidity and control intestinal worms throughout Yemen [14]. This six-year project is implemented by the Yemen Ministry of Public Health and Population (MoPHP) [15] and involves collaborative partnerships with the World Bank, the WHO, the London-based Schistosomiasis Control Initiative (SCI) [16], and the END Fund [17], pharmaceutical donation programs, and several local universities and non-governmental organizations. After treating 4 million children and adults between late 2010 and early 2011, the program was disrupted by civil and political unrest resulting from “the Arab Spring” in 2011. However, activities subsequently re-commenced, and in May/June of 2012, over 2 million people were treated. In 2013, almost 10 million people received treatments for schistosomiasis, intestinal worm infections, or both (Fig. 2).

Though the cause of the success of Yemen's National Schistosomiasis Control Program (YNSCP) cannot be pinpointed to any one factor alone, the mechanisms that fostered the environment that have enabled the program to grow and mature include a system-wide commitment to reduce NTD burden and strong collaborative partnerships. The commitment and drive of the partners working to support NTD control in Yemen (the MoPHP, the YNSCP, the World Bank's YSP, and SCI) have all contributed to the success of the program. Though the in-country NTD teams have faced many challenging situations due to general insecurity throughout many parts of the country, they remain committed to ensuring control efforts targeting schistosomiasis and soil-transmitted helminths reach those in need. Along with the MoPHP's Director General of Disease Control and Surveillance, YNSCP's manager, the in-country WHO NTD coordinator, and other key partners, SCI's resident Program Manager Dr. Dhekra Annuzaili has made an especially important contribution to the country's NTD control efforts. Dr. Annuzaili is responsible for providing technical assistance and programmatic support to the YNSCP within the MoPHP. Dr. Annuzaili is based full-time within the MoPHP and works directly with her counterparts to provide guidance to the disease control officials in the 23 governorates.

In a recent interview with Dr. Annuzaili, her commitment to improving the health of the Yemeni population, even in times of conflict, was made very clear: “It is very risky and very dangerous [in Yemen], but we have to [remain] positive to [ensure] we perform well and reduce [the burden] of these diseases.” She is a public health professional who is successfully supporting disease control efforts in a country where even central-level health authorities have lowered the achievement goals of their health priorities and many international partners have assigned their experts to work remotely [18]. As a woman working in a country with one of the highest gender inequality rankings in the world [19], Dr. Annuzaili has been confronted with many obstacles because of her gender, but her commitment to improving the overall health outcomes in Yemen, to collaborating with her NTD peers and other partners, along with her drive to eliminate NTDs, have enabled her to build strong relationships and contribute to reducing the burden of these devastating diseases.

The achievements of Yemen's NTD control efforts are not solely due to Dr. Annuzaili's personal commitment but also to the contributions of all the partners working to reduce the burden of these diseases of poverty. However, it is important to note that over the course of the many years that Dr. Annuzaili has been working to serve her Yemeni countrymen, she has gathered a wealth of knowledge of how to work in difficult situations. Dr. Annuzaili's tips for NTD peers that are either working in precarious situations or are just launching control efforts include (1) don′t give up—stay committed; (2) prioritize actions; (3) ensure that all parties are responsible for results; (4) search for the truth; (5) social media is a tool that can be useful, but it needs to be managed properly; and (6) be action oriented. These tips, along with an understanding of the importance of strong partner collaboration, can be used by other NTD control programs to strengthen their efforts to reduce the burden of these parasitic and bacterial diseases.

Yemen's MoPHP and NTD partners are now exploring how the successful schistosomiasis and intestinal worm control program can be extended to also include other NTDs, such as onchocerciasis and trachoma. In 2014, the END Fund provided seed funds to support the country's shift from individual case treatment to mass treatment with ivermectin in areas endemic for onchocerciasis—the first step towards the elimination of this disease in Yemen by 2020. Other partners are actively engaged with identifying funding opportunities for trachoma control in the country. The success of Yemen's NTD control efforts provides important lessons for conducting disease control activities in the face of civil unrest, extreme instability, and poverty.
